# Biopsychosocial contexts of timely and adequate prenatal care utilization among women with criminal legal involvement and opioid use disorder

**DOI:** 10.1186/s12889-023-15627-6

**Published:** 2023-04-21

**Authors:** Milan F. Satcher, Martha L. Bruce, Daisy J. Goodman, Sarah E. Lord

**Affiliations:** 1 Department of Community and Family Medicine, Dartmouth Health, Lebanon, NH USA; 2 Center for Technology and Behavioral Health, Geisel School of Medicine, Dartmouth College, Lebanon, NH USA; 3 Department of Psychiatry, Dartmouth Health, Lebanon, NH USA; 4 The Dartmouth Institute, Geisel School of Medicine, Dartmouth College, Hanover, NH USA; 5 Department of Obstetrics-Gynecology, Dartmouth Health, Lebanon, NH USA; 6 Department of Pediatrics, Dartmouth Health, Lebanon, NH USA; 7 Department of Biomedical Data Sciences, Geisel School of Medicine, Dartmouth College, Hanover, NH USA

**Keywords:** Social determinants, Prenatal care, Criminal legal system, Criminal justice, Opioid use disorder, Northern New England

## Abstract

**Objective:**

Pregnant women with criminal legal involvement and opioid use disorder (CL-OUD) living in non-urban regions may be at risk for complex biomedical, psychological, and social barriers to prenatal care and healthy pregnancy. Yet, limited research has explored prenatal care utilization patterns among this subpopulation. This study describes the biopsychosocial factors of pregnant women with a history of criminal legal involvement and opioid use disorder (CL-OUD) associated with timely prenatal care initiation and adequate prenatal care utilization (APNCU).

**Methods:**

Analyses were conducted on a subsample of medical record data from an observational comparative effectiveness study of medication treatment models for pregnant women with diagnosed opioid use disorder (OUD) who received prenatal care in Northern New England between 2015 and 2022. The subsample included women aged ≥ 16 years with documented criminal legal involvement. Analyses included χ^2^, Fisher exact tests, and multiple logistic regression to assess differences in timely prenatal care and APNCU associated with biopsychosocial factors selected by backwards stepwise regression.

**Results:**

Among 317 women with CL-OUD, 203 (64.0%) received timely prenatal care and 174 (54.9%) received adequate care. Timely prenatal care was associated with having two or three prior pregnancies (aOR 2.37, 95% CI 1.07–5.20), receiving buprenorphine at care initiation (aOR 1.85, 95% CI 1.01–3.41), having stable housing (aOR 2.49, 95% CI 1.41–4.41), and being mandated to court diversion (aOR 4.06, 95% CI 1.54–10.7) or community supervision (aOR 2.05, 95% CI 1.16–3.63). APNCU was associated with having a pregnancy-related medical condition (aOR 2.17, 95% CI 1.27–3.71), receiving MOUD throughout the entire prenatal care period (aOR 3.40, 95% CI 1.45–7.94), having a higher number of psychiatric diagnoses (aOR 1.35, 95% CI 1.07–1.70), attending a rurally-located prenatal care practice (aOR 2.14, 95% CI 1.22–3.76), having stable housing (aOR 1.94, 95% CI 1.06–3.54), and being mandated to court diversion (aOR 3.11, 95% CI 1.19–8.15).

**Conclusion:**

While not causal, results suggest that timely and adequate prenatal care among women with CL-OUD may be supported by OUD treatment, comorbid indications for care, stable access to social resources, and maintained residence in the community (i.e., community-based alternatives to incarceration).

## Introduction

Pregnant women in rural and small metropolitan regions may be disproportionately affected by criminal legal involvement and opioid use disorder (CL-OUD). The highest prevalence and incidence of maternal opioid use has occurred in regions like Northern New England (NNE), which consists of predominantly rural and a few small metropolitan areas spanning Maine, New Hampshire, and Vermont. In NNE, opioid use disorder (OUD) affects, on average, 5–8% of pregnancies, with considerable variability; the prevalence of Neonatal Abstinence Syndrome is 3–4 times higher than the national prevalence, and drug overdose is the leading cause of pregnancy-related death [1–3]. Consistent with national trends, incarceration rates in NNE’s rural counties have outpaced those of urban counties, likely in part driven by the high prevalence of OUD and lower availability of both OUD treatments and social resources in non-urban locales [4–8].

Pregnant women have a higher risk of arrest for a drug-related offense and receive harsher sentencing upon conviction than non-pregnant people [9]. In NNE, only Maine legally requires reporting of diagnosed or suspected prenatal substance use [10]. However, women have been prosecuted for prenatal substance use in NH [11]. In VT, civil statutes and agency policies advise the reporting of prenatal exposure to controlled substances in the third trimester and include prenatal exposure in the definition of child abuse [12]. Pregnant women with CL-OUD understand their unique vulnerability to the criminal legal system and appropriately recognize medicalized stigma of OUD and criminal legal history as a mediator; more than 40% of legal cases against pregnant women in the United States originate from healthcare reports to legal entities and child protective services [9]. Whether experienced during pregnancy or prior, an experience of criminal legal involvement can impart risks of criminalization, termination of parental rights, and structural barriers (e.g., collateral consequences of convictions) to engaging in prenatal care [13–15].

Pregnant women with CL-OUD living in non-urban areas face additional complex biomedical, psychological, and social barriers to prenatal care. Prior studies show that reproductive-aged women with criminal legal experience, OUD, and rural residence are more likely to have poor baseline health and unstable social resources than the general population [16–18]. In pregnancy, they may be more likely to experience perinatal complications, exacerbation of underlying medical and psychiatric comorbidities, and heightened interpersonal victimization [19, 20]. Limited research has explored the prenatal care utilization patterns of pregnant women with CL-OUD in non-urban environments. The few prior studies have described late initiation of prenatal care and poor retention in prenatal care among community-based women with a history of personal or partner incarceration, OUD, and rural residence [21–25]. To address this literature gap, this study describes the biopsychosocial portraits of pregnant women living at the intersection of CL-OUD in the largely rural NNE region and aims to identify biomedical, psychological, and social correlates of timely and adequate prenatal care in this subpopulation.

## Methods

### Data source and sampling method

This study is a sub-analysis of medical record data collected as part of an observational comparative effectiveness study comparing maternal and infant outcomes for pregnant women with OUD who received MOUD within the context of maternity care versus referral-based specialty addiction treatment (henceforth referred to as the “parent study”). The parent dataset represents a convenience sample of the prenatal medical records of 1,795 pregnant women with OUD aged 16 years and older who had at least one prenatal care visit documented at partner maternity care settings and received prenatal care between 2015 and 2022. Each participant corresponds to one pregnancy episode. Data were collected from 21 outpatient maternity care sites (i.e., obstetrics-gynecology, family medicine, and midwifery practices) located in academic and community health settings throughout NNE. These data included documented history of criminal legal involvement, sociodemographic characteristics, pre-pregnancy conditions, pregnancy-related medical history, mental health history, substance use conditions and treatments, obstetric course, perinatal health service utilization, and medical and psychosocial maternal outcomes. A trained team of clinical researchers manually extracted the medical record data using a detailed, standardized data collection template. Trained study personnel input the data into the secure, HIPAA-compliant Research Electronic Data Capture (REDCap) tool hosted at Dartmouth College [26, 27]. The Institutional Review Board at Dartmouth College and partner institutions approved the study procedures.

### Inclusion/exclusion criteria

The sample for the sub-analysis was restricted to patients with prenatal record documentation of: (1) current or history of criminal legal involvement, (2) gestational age (in weeks) at which prenatal care was initiated, and (3) delivery ≥ 20 weeks gestation. The definition of criminal legal involvement included incarceration, parole, probation, location monitoring, treatment court, other court diversion program, arrest, and/or awaiting a court hearing.

### Outcome measures

The two primary outcomes were the (1) timing of prenatal care initiation and (2) adequacy of prenatal care utilization (APNCU) as assessed by the Kotelchuck Index [28]. Timely prenatal care initiation is defined as the first prenatal care visit occurring during the first trimester (i.e., < 14 weeks of gestation per ACOG guidelines) [29]. Late prenatal care initiation is defined as the first prenatal care visit occurring after the first trimester (i.e., ≥ 14 weeks of gestation). APNCU consists of the month prenatal care was initiated and the percent of ACOG-recommended prenatal care visits that were attended during the pregnancy period, adjusted by the gestational age at prenatal care initiation and delivery (see Table 1). As operationalized in prior studies, adequate prenatal care is defined as prenatal care initiated less than 4 months and ≥ 80% prenatal visits attended, and inadequate prenatal care is defined as < 80% prenatal care visits attended, regardless of timing of the first prenatal visit [20, 30]. Gestational age is defined as the number of completed weeks of gestation (e.g., 13 weeks 5 days equals 13 weeks) as documented in the prenatal record. The study team confirmed gestational age by reviewing prenatal ultrasound(s), when available. The study team made every effort to obtain medical records and correct the gestational age at prenatal care initiation for participants who received prenatal care prior to transfer to partner sites.

**Table 1:** Adequacy of prenatal care utilization (Kotelchuck Index) [28]

Kotelchuck Index Categories	Definition	Binary Categories
Adequate Plus	1st prenatal visit ≤ 4 months and ≥ 110% prenatal visits	Adequate Care
Adequate	1st prenatal visit ≤ 4 months and 80–109% prenatal visits	
Intermediate	1st prenatal visit ≤ 4 months and 50–79% prenatal visits	Inadequate care
Inadequate	1st prenatal visit > 4 months *-or-* < 50% prenatal visits	

### Biomedical factors

Biomedical variables included age, gravidity, parity, chronic pre-pregnancy medical conditions, pregnancy-related medical conditions, nicotine use, OUD severity, documented history of injection drug use, lab-determined hepatitis C status, type of MOUD at prenatal care initiation, and duration of MOUD in the prenatal care period. Age was coded as continuous variables. Gravidity and parity were categorized as “first,” “second or third,” and “fourth or greater” pregnancy and delivery, respectively. Chronic medical condition and pregnancy-related medical condition were categorized as “yes” or “no.” Chronic medical conditions included chronic hypertension, cardiac disease, pulmonary conditions, renal disorders, metabolic disorders, chronic infectious disease, and genetic disorders present prior to the pregnancy. Pregnancy-related medical conditions included hyperemesis gravidarum, gestational diabetes, gestational hypertension, preeclampsia, cholestasis of pregnancy, hypothyroidism of pregnancy, cervical insufficiency, intrauterine growth restriction, oligo- and polyhydramnios, second and third trimester vaginal bleeding, placental previa, and placental abruption. Hepatitis C diagnosis was categorized as “active infection,” “history of infection,” or “none.” Type of MOUD at prenatal care initiation was defined as “buprenorphine,” “methadone,” or “none documented.” Duration of MOUD was defined as “entire prenatal care period,” “documented MOUD interruption,” and “no MOUD documented.” OUD severity was coded as “mild,” “moderate,” or “severe.”

### Psychological factors

Psychological variables included the documented diagnosis of mood disorders and anxiety disorders, coded as “yes” if documented. History of interpersonal trauma was coded as “yes” or “no.” A continuous variable was created representing number of documented psychiatric diagnoses.

### Social factors

Social variables included race, ethnicity, marital status, education, employment during pregnancy, health insurance type, prenatal care practice location, transportation to appointments, number of live children, legal parenting status, and documented history of child protective service involvement. The operationalization of these variables is shown in Table 2. Criminal legal experiences frequently overlap by nature of the U.S. judicial process. Due to the cross-sectional nature of the data, we were unable to cleanly isolate the timing of distinct criminal legal experiences. Therefore, patients were categorized by the most intensive criminal legal experience documented, which was operationalized as “incarceration [includes jail, prison, and undefined ‘incarceration’],” “court diversion program [includes treatment court and other diversion],” or “community supervision [includes probation, parole, location monitoring, and pretrial supervision].” Stable housing was defined as “yes,” if there was documentation of housing by family, friends, or in one’s independent residence without shelter experience, or “no,” meaning the patient was unhoused or stayed in shelters.

**Table 2:** Sample characteristics and prenatal care utilization among pregnant women with CL-OUD

	**Total**	**Timely Prenatal Care**	**Late Prenatal Care**	***p* value**^a^	**Adequate Prenatal Care**	**Inadequate Prenatal Care**	***p* value**^a^
	*N* = 317	*N* = 203 (64.0%)	*N* = 114 (36.0%)		*N* = 174 (54.9%)	*N* = 143 (45.1%)	
Age, years ^b^	28.1 ± 4.5 (R: 18–42)	28.0 ± 4.4	28.2 ± 4.8	0.67	28.0 ± 4.6	28.2 ± 4.5	0.63
Gravidity ^c^				0.12			0.28
1 (First Pregnancy)	42 (13.2%)	23 (11.3%)	19 (16.7%)		24 (13.8%)	18 (12.6%)	
2–3	122 (38.5%)	86 (42.4%)	36 (31.6%)		73 (42.0%)	49 (34.3%)	
4 +	153 (48.3%)	94 (46.3%)	59 (51.8%)		77 (44.3%)	76 (53.1%)	
Parity				0.76			0.42
1 (First Delivery)	75 (23.9%)	48 (23.8%)	27 (24.1%)		46 (26.7%)	29 (20.4%)	
2–3	170 (54.1%)	112 (55.4%)	58 (51.8%)		89 (51.7%)	81 (57.0%)	
4 +	69 (22.0%)	42 (20.8%)	27 (24.1%)		37 (21.5%)	32 (22.5%)	
Chronic Medical Condition ^c^				0.38			0.15
No	80 (25.2%)	48 (23.6%)	32 (28.1%)		38 (21.8%)	42 (29.4%)	
Yes	237 (74.8%)	155 (76.4%)	82 (71.9%)		136 (78.2%)	101 (70.6%)	
Pregnancy-Related Medical Condition ^c^							0.001^‡^
No	176 (55.5%)	-	-		82 (47.1%)	94 (65.7%)	
Yes	141 (44.5%)	-	-		92 (52.9%)	49 (34.3%)	
Nicotine Use in Pregnancy				0.36			0.67
No	48 (15.2%)	28 (13.9%)	20 (17.7%)		25 (14.4%)	23 (16.2%)	
Yes	267 (84.8%)	174 (86.1%)	93 (82.3%)		148 (85.6%)	119 (83.8%)	
OUD Severity ^d^				0.67			0.27
Mild	8 (5.0%)	6 (5.5%)	2 (4.0%)		7 (7.1%)	1 (1.6%)	
Moderate	37 (23.1%)	23 (20.9%)	14 (28.0%)		21 (21.4%)	16 (25.8%)	
Severe	115 (71.9%)	81 (73.6%)	34 (68.0%)		70 (71.4%)	45 (72.6%)	
History of Injection Drug Use ^c^				0.41			0.10
No	91 (28.8%)	55 (27.2%)	36 (31.6%)		43 (24.9%)	48 (33.6%)	
Yes	225 (71.2%)	147 (72.8%)	78 (68.4%)		130 (75.1%)	95 (66.4%)	
Hepatitis C Diagnosis ^c^				0.21			0.17
None	130 (42.3%)	86 (43.4%)	44 (40.4%)		69 (41.1%)	61 (43.9%)	
History of Infection	63 (20.5%)	45 (22.7%)	18 (16.5%)		41 (24.4%)	22 (15.8%)	
Active Infection	114 (37.1%)	67 (33.8%)	47 (43.1%)		58 (34.5%)	56 (40.3%)	
MOUD at Prenatal Care Initiation ^c^				0.16			
Buprenorphine	174 (55.8%)	119 (59.5%)	55 (49.1%)		-	-	
Methadone	58 (18.6%)	36 (18.0%)	22 (19.6%)		-	-	
None documented	80 (25.6%)	45 (22.5%)	35 (31.2%)		-	-	
Duration of MOUD ^c^							0.003^‡^
Entire Prenatal Care Period	213 (68.9%)	-	-		130 (76.5%)	83 (59.7%)	
Documented MOUD Interruption	38 (12.3%)	-	-		13 (7.6%)	25 (18.0%)	
No MOUD documented	58 (18.8%)	-	-		27 (15.9%)	31 (22.3%)	
Mood Disorder				0.56			0.32
No	61 (19.2%)	37 (18.2%)	24 (21.1%)		30 (17.2%)	31 (21.7%)	
Yes	256 (80.8%)	166 (81.8%)	90 (78.9%)		144 (82.8%)	112 (78.3%)	
Anxiety Disorder				0.26			0.34
No	69 (21.8%)	40 (19.7%)	29 (25.4%)		34 (19.5%)	35 (24.5%)	
Yes	248 (78.2%)	163 (80.3%)	85 (74.6%)		140 (80.5%)	108 (75.5%)	
History of any Interpersonal Trauma ^c^				0.05			0.19
No	139 (45.0%)	80 (40.8%)	59 (52.2%)		69 (41.6%)	70 (49.0%)	
Yes	170 (55.0%)	116 (59.2%)	54 (47.8%)		97 (58.4%)	73 (51.0%)	
History of Physical Trauma ^d^				0.33			0.44
No	177 (64.4%)	105 (62.1%)	72 (67.9%)		89 (62.2%)	88 (66.7%)	
Yes	98 (35.6%)	64 (37.9%)	34 (32.1%)		54 (37.8%)	44 (33.3%)	
History of Sexual Trauma ^d^				0.03^‡^			0.41
No	176 (63.3%)	101 (58.4%)	75 (71.4%)		91 (61.1%)	85 (65.9%)	
Yes	102 (36.7%)	72 (41.6%)	30 (28.6%)		58 (38.9%)	44 (34.1%)	
Number of Psychiatric Diagnoses ^b, c^	2.1 ± 1.2 (R: 0–5)	2.2 ± 1.2	2.0 ± 1.3	0.43	2.3 ± 1.2	2.0 ± 1.1	0.03^‡^
Race ^e^				0.40			0.20
Black or African American	2 (0.6%)	1 (0.5%)	1 (0.9%)		0 (0.0%)	2 (1.5%)	
American Indian or Alaskan Native	3 (1.0%)	3 (1.5%)	0 (0.0%)		3 (1.8%)	0 (0.0%)	
Asian	0 (0.0%)	-	-		-	-	
Native Hawaiian or Pacific Islander	1 (0.3%)	0 (0.0%)	1 (0.9%)		1 (0.6%)	0 (0.0%)	
White	300 (97.4%)	195 (97.5%)	105 (97.2%)		166 (97.1%)	134 (97.8%)	
Multiracial	2 (0.6%)	1 (0.5%)	1 (0.9%)		1 (0.6%)	1 (0.7%)	
Ethnicity ^e^				0.50			1.00
Hispanic/Latina	9 (2.9%)	7 (3.6%)	2 (1.8%)		5 (3.0%)	4 (2.9%)	
Non-Hispanic/Latina	297 (97.1%)	190 (96.4%)	107 (98.2%)		162 (97.0%)	135 (97.1%)	
Marital Status				0.33			0.64
Single	76 (25.0%)	53 (26.8%)	23 (21.7%)		44 (26.0%)	32 (23.7%)	
Married or Partnered	228 (75.0%)	145 (73.2%)	83 (78.3%)		125 (74.0%)	103 (76.3%)	
Stable Housing Source ^c^				< 0.001^‡^			0.002^‡^
No	82 (26.8%)	37 (18.9%)	45 (40.9%)		34 (19.8%)	48 (35.8%)	
Yes	224 (73.2%)	159 (81.1%)	65 (59.1%)		138 (80.2%)	86 (64.2%)	
Education ^d^				0.77			0.63
Less than secondary school	53 (26.9%)	34 (25.4%)	19 (30.2%)		31 (27.7%)	22 (25.9%)	
Secondary school or GED completed	90 (45.7%)	63 (47.0%)	27 (42.9%)		48 (42.9%)	42 (49.4%)	
Tertiary, partial and completed	54 (27.4%)	37 (27.6%)	17 (27.0%)		33 (29.5%)	21 (24.7%)	
Employment ^c^				0.14			0.34
Any unemployment	211 (73.8%)	138 (71.1%)	73 (79.3%)		119 (71.7%)	92 (76.7%)	
Employed only	75 (26.2%)	56 (28.9%)	19 (20.7%)		47 (28.3%)	28 (23.3%)	
Health Insurance ^e^				0.15			0.48
Private only	7 (2.2%)	3 (1.5%)	4 (3.6%)		3 (1.7%)	4 (2.9%)	
Private and Public	6 (1.9%)	6 (3.0%)	0 (0.0%)		5 (2.9%)	1 (0.7%)	
Public (i.e., Medicaid, State Plan)	284 (91.0%)	181 (90.0%)	103 (92.8%)		155 (90.1%)	129 (92.1%)	
Uninsured	15 (4.8%)	11 (5.5%)	4 (3.6%)		9 (5.2%)	6 (4.3%)	
Prenatal Care Location ^c^				0.02^‡^			< 0.001^‡^
Non-rural	132 (41.6%)	75 (36.9%)	57 (50.0%)		58 (33.3%)	74 (51.7%)	
Rural	185 (58.4%)	128 (63.1%)	57 (50.0%)		116 (66.7%)	69 (48.3%)	
Utilized own transportation ^d^				0.19			0.08
No	145 (71.8%)	89 (68.5%)	56 (77.8%)		78 (66.7%)	67 (78.8%)	
Yes	57 (28.2%)	41 (31.5%)	16 (22.2%)		39 (33.3%)	18 (21.2%)	
Transportation by family or friends ^d^				0.17			0.98
No	119 (58.9%)	72 (55.4%)	47 (65.3%)		69 (59.0%)	50 (58.8%)	
Yes	83 (41.1%)	58 (44.6%)	25 (34.7%)		48 (41.0%)	35 (41.2%)	
Utilized publicly subsidized transportation ^d^				0.02^‡^			0.04^‡^
No	117 (57.9%)	83 (63.8%)	34 (47.2%)		75 (64.1%)	42 (49.4%)	
Yes	85 (42.1%)	47 (36.2%)	38 (52.8%)		42 (35.9%)	43 (50.6%)	
Most Intensive Legal Involvement ^c^				< 0.001^‡^			0.001^‡^
Incarceration	151 (50.3%)	80 (41.5%)	71 (66.4%)		70 (41.9%)	81 (60.9%)	
Court Diversion Program	42 (14.0%)	35 (18.1%)	7 (6.5%)		32 (19.2%)	10 (7.5%)	
Community Supervision	107 (35.7%)	78 (40.4%)	29 (27.1%)		65 (38.9%)	42 (31.6%)	
Number of live children				0.38			0.36
0	83 (26.2%)	53 (26.1%)	30 (26.3%)		51 (29.3%)	32 (22.4%)	
1	97 (30.6%)	68 (33.5%)	29 (25.4%)		54 (31.0%)	43 (30.1%)	
2	70 (22.1%)	40 (19.7%)	30 (26.3%)		33 (19.0%)	37 (25.9%)	
3 +	67 (21.1%)	42 (20.7%)	25 (21.9%)		36 (20.7%)	31 (21.7%)	
Parenting Status ^d^				0.01^‡^			0.68
No custody of older children	147 (63.4%)	86 (57.3%)	61 (74.4%)		77 (62.1%)	70 (64.8%)	
Current custody of older children	85 (36.6%)	64 (42.7%)	21 (25.6%)		47 (37.9%)	38 (35.2%)	
History of Child Protective Service Involvement ^d^				1.00			0.53
No	62 (32.1%)	41 (32.3%)	21 (31.8%)		39 (33.9%)	23 (29.5%)	
Yes	131 (67.9%)	86 (67.7%)	45 (68.2%)		76 (66.1%)	55 (70.5%)	

^‡^ Statistically significant, *p*-value < 0.05

^a^*p*-values were obtained by Student t-test for continuous variables, χ^2^ test, or Fisher exact test for small samples for categorical variables

^b^ Mean, standard deviation, and range values calculated for continuous variables

^c^ Variable considered for inclusion in multivariable analysis when *p*-value < 0.25 and < 10% missing data

^d^ Variable has more than 10% missing data

^e^ Variable excluded from multivariable analysis due to lack of sufficient cell variability

### Statistical analysis

Bivariate analyses with individual-level biopsychosocial characteristics were conducted to identify variables for inclusion in the backward stepwise regression model for each outcome. Bivariate comparisons were performed using the student’s t-test for continuous variables and the χ^2^ test or Fisher exact test for small samples for categorical variables. Variables with less than 10% missing observations and *p* < 0.25 in bivariate analysis were selected for inclusion in the backward stepwise models, which then removed variables with *p* ≥ 0.25 and added those with *p* < 0.1 to build the full multivariable logistic regression model [31]. Odds ratios (OR) with 95% confidence intervals (CI) were calculated to determine the odds of receiving (1) timely adequate prenatal care and (2) adequate prenatal care utilization associated with selected biomedical, psychological, and social characteristics. Two-tailed *p* values were calculated, and significance was set at *p* < 0.05. The documented prenatal visit period (2015–2022) overlapped with the COVID-19 pandemic, during which states attempted to reduce the number of persons entering and maintained within the criminal legal system. Sensitivity analyses were conducted using the same model building strategy to re-examine outcomes among women who initiated prenatal care prior to the pandemic (2015–2019). Analyses were performed using Stata statistical software, version 16 [32].

## Results

### Data quality and sample characteristics

A total of 329 women had documented history of criminal legal involvement, of which 317 met the full inclusion criteria for this analysis. Among the sample, most biomedical, psychological, and social characteristics were well-documented in the medical records, except for OUD severity (*n* = 157, 49.5% missing), transportation (115, 36.3% missing), education (*n* = 120, 37.9% missing), and history of child protective service involvement (*n* = 124, 39.1%). Among all variables considered, the minimum missing observations was 0.3% (*n* = 1) missing and maximum was 49.5% (*n* = 157) missing.

Table 2 shows the biopsychosocial characteristics of the sample. The mean age was 28.1 years. The sample included forty-two (13.2%) first pregnancies and 75 (23.9%) first deliveries. Most women had a chronic medical condition (*n* = 237, 74.8%). At prenatal care initiation, women more commonly were taking buprenorphine (*n* = 174, 55.8%) than methadone (*n* = 58, 18.6%). Two-thirds received MOUD during the entire prenatal care period (*n* = 213, 68.9%), while 38 (12.3%) were documented as receiving MOUD during part of the prenatal care period and 58 (18.8%) had no documentation of MOUD. A high proportion of the sample experienced psychological morbidity, including mood disorders (*n* = 256, 78.2%) and anxiety disorders (*n* = 248, 78.2%); 42 women (12.1%) did not have a documented mental health condition. Trauma was common, with 170 (55.0%) having a documented history of interpersonal trauma, including physical (*n* = 98, 35.6%) and/or sexual (*n* = 102, 36.7%) trauma.

Women in this sample were predominantly White (*n* = 300, 97.4%) and non-Hispanic (*n* = 297, 97.1%). Most women were married or partnered (*n* = 228, 75.0%), stably housed (*n* = 224, 73.2%), unemployed (*n* = 211, 73.8%), received public health insurance coverage (i.e., Medicaid or state health plans; *n* = 284, 91.0%), and established prenatal care in rural practices (*n* = 185, 58.4%). Most women also had a documented history of injection drug use (*n* = 209, 72.6%) and had experienced incarceration (*n* = 138, 50.5%). Among the women with at least one living child (*n* = 234, 73.8% of sample), 36.6% (*n* = 85) of them had custody and 63.4% (*n* = 147) did not. Only 193 (60.9%) records documented whether or not child protective services were involved, among which 131 (67.9%) had experienced child protective service involvement.

### Prenatal care utilization

**Fig. 1:**
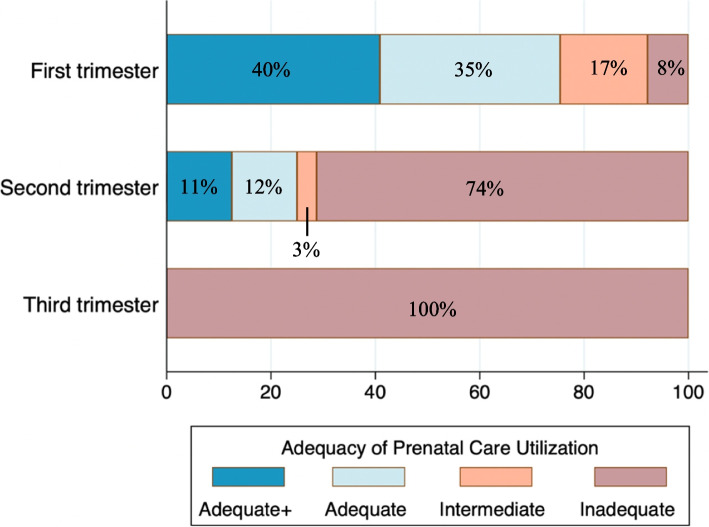
Timing and adequacy of prenatal care utilization

The median gestational age at prenatal care initiation was 11 weeks (interquartile range: 8, 16; range: 1–40) and median gestational age at delivery was 39 weeks (interquartile range: 37, 40; range: 25–42). Most women (*n* = 203, 64.0%) initiated prenatal care in the first trimester (i.e., timely). Prenatal care was initiated in the second and third trimester (i.e., late) by 92 (29.0%) and 22 (7.0%) of women, respectively. Ninety-two (29.0%) women received adequate plus and 82 (25.9%) received adequate care, which collectively represent “adequate care” for this study. Thirty-seven (11.7%) women received intermediate care and 106 (33.4%) received inadequate care, which collectively represent “inadequate care.” Figure 1 shows the distribution of prenatal care utilization for this sample.

### Timing of prenatal care initiation

Table 2 depicts the associations of prenatal care initiation with sample characteristics. Compared to late care initiation, timely care initiation was more likely among women with two or three prior pregnancies (42.4% vs. 31.6%), a history of hepatitis C (22.7% vs. 16.5%), buprenorphine at pregnancy initiation (59.5% vs. 49.1%), trauma history (59.2% vs. 47.8%), stable housing (81.1% vs. 59.1%), employment (28.9% vs. 20.7%), rural prenatal care location (63.1% vs. 50.0%), court diversion (18.1% vs. 6.5%), or community supervision (40.4% vs. 27.1%). All the above variables met statistical criteria for entry into the backward stepwise logistic regression model.

Table 3 shows the logistic regression of timely prenatal care and biopsychosocial correlates. Compared to late prenatal care, the odds of timely prenatal care were significantly higher among women with two or three prior pregnancies (aOR 2.37, 95% CI 1.07–5.20; reference: first pregnancy), buprenorphine at prenatal care initiation (aOR 1.85, 95% CI 1.01, 3.41; reference: no MOUD), stable housing (aOR 2.49, 95% CI 1.41, 4.41; reference: unstable housing), and experience in court diversion (aOR 4.06, 95% CI 1.54–10.7; reference: incarceration) or community supervision (aOR 2.05, 95% CI 1.16–3.63; reference: incarceration). The Hosmer–Lemeshow goodness-of-fit failed to reject the null (*p* = 0.80), which supports the model as well-fit. The area under the Receiver Operating Characteristic curve (0.71) indicated that the model performed significantly better than chance.

**Table 3:** Biopsychosocial correlates of timely prenatal care^*^N = 285

	**Crude Odds Ratio** **(95% CI)**	***p* value**	**Adjusted Odds Ratio** **(95% CI)**	***p* value**
*Biomedical Factors*				
Gravidity				
1 (First Pregnancy)	1 [Reference]		1 [Reference]	
2–3	1.97 (0.96–4.06)	0.07	2.37 (1.07–5.20)	0.03^‡^
4 +	1.32 (0.66–2.62)	0.44	1.66 (0.78–3.52)	0.19
MOUD at Prenatal Care Initiation				
None documented	1 [Reference]		1 [Reference]	
Buprenorphine	1.68 (0.98–2.90)	0.06	1.85 (1.01–3.41)	0.048^‡^
Methadone	1.27 (0.64–2.54)	0.49	1.63 (0.75–3.53)	0.21
*Social Factors*				
Stable Housing Source				
No	1 [Reference]		1 [Reference]	
Yes	5.48 (2.83–10.64)	< 0.001^‡^	2.49 (1.41–4.41)	0.002^‡^
Most Intensive Legal Involvement				
Incarceration	1 [Reference]		1 [Reference]	
Court Diversion Program	4.90 (1.93–12.4)	0.001^‡^	4.06 (1.54–10.73)	0.005^‡^
Community Supervision	2.83 (1.59–5.03)	< 0.001^‡^	2.05 (1.16–3.63)	0.01^‡^

^*^Hosmer–Lemeshow *p*-value: 0.80. Area under ROC curve: 0.7082

^‡^ Statistically significant, *p*-value < 0.05

### Adequacy of prenatal care utilization

Table 2 describes the adequacy of prenatal care utilization by sample characteristics. Compared to inadequate utilization, adequate prenatal care utilization was more likely among women who had a chronic medical condition (78.2% vs. 70.6%), pregnancy-related medical condition (52.9% vs. 34.3%), history of injection drug use (75.1% vs. 66.4%), history of hepatitis C infection (24.4% vs. 15.8%), MOUD during the entire prenatal care period (76.5% vs. 59.7%), trauma history (58.4% vs. 51.0%), higher burden of psychiatric diagnoses (2.3 vs. 2.0 diagnoses), stable housing (80.2% vs. 64.2%), rural prenatal care location (66.7% vs. 48.3%), court diversion (19.2% vs. 7.5%), and community supervision (38.9% vs. 31.6%). All the aforementioned variables met statistical criteria for entry into the backward stepwise logistic regression model.

Table 4 shows the logistic regression of the APNCU and associated biopsychosocial correlates. Compared to inadequate prenatal care utilization, the odds of adequate care were significantly higher among women with a pregnancy-related medical condition (aOR 2.17, 95% CI 1.27–3.71; reference: no pregnancy-related condition), MOUD during the entire prenatal care period (aOR 3.40, 95% CI 1.45–7.94; reference: MOUD during part of prenatal care period), psychiatric diagnoses (35% increased odds for every additional diagnosis; 95% CI 1.07–1.70), rural prenatal practice location (aOR 2.14, 95% CI 1.22–3.76; reference: non-rural practice), stable housing (aOR 1.94, 95% CI 1.06–3.54; reference: unstable housing), and experience in court diversion (aOR 3.11, 95% CI 1.19–8.15; reference: incarceration). The Hosmer–Lemeshow goodness-of-fit failed to reject the null (*p* = 0.98), which supports the model as well-fit. The area under the Receiver Operating Characteristic curve (0.75) indicated that the model performed significantly better than chance.

**Table 4:** Biopsychosocial correlates of adequate prenatal care^*^N = 282

	**Crude Odds Ratio** **(95% CI)**	***p* value**	**Adjusted Odds Ratio** **(95% CI)**	***p* value**
*Biomedical Factors*				
Pregnancy-Related Medical Condition				
No	1 [Reference]		1 [Reference]	
Yes	2.02 (1.25–3.25)	0.004^‡^	2.17 (1.27–3.71)	0.004^‡^
History of Injection Drug Use		0.10		0.12
No	1 [Reference]		1 [Reference]	
Yes	1.54 (0.92–2.60)		1.60 (0.88–2.90)	
MOUD during Prenatal Care Period				
Entire Prenatal Care Period	3.37 (1.63–6.96)	0.001^‡^	3.40 (1.45–7.94)	0.005^‡^
Part of Prenatal Care Period	1 [Reference]		1 [Reference]	
No MOUD documented	1.71 (0.73–4.05)	0.22	2.16 (0.80–5.86)	0.13
*Psychological Factors*				
Number of Psychiatric Diagnoses	1.24 (1.02–1.52)	0.03^‡^	1.35 (1.07–1.70)	0.01^‡^
*Social Factors*				
Prenatal Care Practice Location				
Non-rural	1 [Reference]		1 [Reference]	
Rural	2.28 (1.42–3.68)	0.001^‡^	2.14 (1.22–3.76)	0.01^‡^
Stable Housing Source				
No	1 [Reference]		1 [Reference]	
Yes	4.06 (2.09–7.89)	< 0.001^‡^	1.94 (1.06–3.54)	0.03^‡^
Most Intensive Legal Involvement				
Incarceration	1 [Reference]		1 [Reference]	
Court Diversion Program	3.85 (1.70–8.72)	0.001^‡^	3.11 (1.19–8.15)	0.02^‡^
Community Supervision	2.02 (1.18–3.44)	0.01^‡^	1.25 (0.71–2.20)	0.44

^*^Hosmer–Lemeshow *p*-value: 0.98. Area under ROC curve: 0.7521

^‡^ Statistically significant, *p*-value < 0.05

### Sensitivity analyses

Timely prenatal care remained significantly associated with gravidity of 2–3 (aOR 2.71, 95% CI 1.15–6.40; reference: first pregnancy), both forms of MOUD (Buprenorphine aOR 2.14, 95% CI 1.09–4.19; Methadone aOR 2.48, 95% CI 1.02–6.03; reference: no MOUD), stable housing (aOR 2.93, 95% CI 1.53–5.60; reference: unstable housing), court diversion (aOR 5.00, 95% CI 1.56–16.1; reference: incarceration), and community supervision (aOR 2.24, 95% CI 1.17–4.32; reference: incarceration). Adequate prenatal care remained significantly associated with pregnancy-related medical morbidity (aOR 2.33, 95% CI 1.29–4.22; reference: none), MOUD received throughout entire prenatal care period (aOR 3.53, 95% CI 1.40–8.89; reference: part of prenatal care period), number of psychiatric diagnoses (37% increased odds for every additional diagnosis, 95% CI 1.07, 1.76), rural prenatal care location (aOR 1.84, 95% CI 1.00–3.41; reference: urban), and court diversion (aOR 3.53, 95% CI 1.19–10.5; reference: incarceration), adjusted by stable housing and history of injection drug use. Overall, the team considered these results as not substantively different from the primary results.

## Discussion

This study described the prenatal contexts and care disparities of a sample of women with CL-OUD in the largely rural and small metropolitan region of NNE. The sample experienced a high burden of medical morbidity, psychiatric illness, and structural vulnerability, consistent with prior research [16–19]. Despite living in a region that leads the nation in prenatal care utilization, documentation in medical records indicated that timely and adequate prenatal care among the sample were at least 22% and 28% less prevalent, respectively, than the general NNE population [33]. Compared to a national analysis, the prenatal care distribution by trimester for our sample (Fig. 1) showed a higher proportion of inadequate care in both the first and second trimesters [34]. Given the recognized benefits of prenatal care for improving pregnancy outcomes, these disparities are concerning. While causality cannot be determined, our results suggest several biomedical, psychological, and social factors may facilitate prenatal care utilization among women with CL-OUD in non-urban communities and should therefore be the focus of efforts to improve access to care.

### MOUD

Our findings suggest that early and continued MOUD in pregnancy may support prenatal care utilization, consistent with prior findings of improved maternal and child health outcomes with MOUD treatment in pregnancy [35, 36]. Pregnancy may motivate women to seek early prenatal care and OUD treatment [37]. Alternatively, our findings may reflect the clinical and psychosocial outcomes of MOUD initiated prior to conception. Compared to methadone, buprenorphine is more accessible in rural areas and may be associated with less stigma, which may account for our findings [38–40]. Prioritizing early access to MOUD for reproductive-aged women with CL-OUD may improve their baseline physical and psychosocial health and increase access to family planning, early identification of pregnancy, and timely, sustained connection to prenatal care, with positive implications for maternal and child health outcomes.

### Prior pregnancy and comorbid medical/psychological conditions

Prior experience of pregnancy may support early pregnancy identification and timely prenatal care initiation. Active symptoms of psychiatric or pregnancy-related medical conditions may drive patients to utilize care to manage symptoms and prevent adverse pregnancy outcomes. Given that most of the sample was multiparous and that pregnancy-related conditions have a risk of recurrence, our findings may reflect recurrent conditions that lead to increased care engagement among an experienced cohort [41–43]. In an ideal care system, diagnosis of a medical or psychiatric condition should trigger a cascade of clinical case management that exceeds routine prenatal care and results in more frequent visits [29]. Our results may reflect that the study’s prenatal care systems appropriately engaged patients with high-risk conditions. Future research should examine the appropriateness of using the Kotelchuck Index as an indicator of equitable care responses and how to operationalize APNCU for high-risk pregnancies.

### Rural prenatal care location

Women with CL-OUD in rural regions often lack access to a functional or registered car; many also have lost or suspended driver’s licenses associated with sentencing conditions or incarceration and lack access to public transit between rural and urban areas [44, 45]. Previous research has found lower prenatal care use at non-local care sites [46, 47]. Our findings suggest that increasing rural prenatal care (i.e., local access to care) may contribute to prenatal care utilization.

### Stable housing

Our results suggest that economic resources (i.e., independent housing) and social capital (i.e., family/friends who provide housing) facilitate timely and adequate prenatal care. While research has shown that some family and social connections can be stigmatizing and undermine the development of a maternal identity among women with CL-OUD, positive connections provide psychosocial support, promote healthy behaviors (e.g., appointment attendance), and supply tangible resources (e.g., housing, transportation, food, childcare) [48–50]. Stable housing also increases access to contingency-based social resources that support care utilization; for example, Medicaid-sponsored rides to medical appointments requires a home address and must be scheduled at least 2 days in advance, which effectively excludes women with unstable housing [51]. Policies and communities should invest in pathways to promote stable housing and healthy social capital, and to remove barriers to service access (e.g., housing requirement for transportation) for women with CL-OUD.

### Community-based alternatives to incarceration

Compared to incarceration, court diversion and community supervision may better enable timely linkage to prenatal care by maintaining stable residence in the community and stable access to health insurance and care providers. Both also mandate contact with medical providers, legal income, and stable housing; these resources may support prenatal care linkage and retention [52–54]. Treatment courts in NNE support and supervise the provision of MOUD (including buprenorphine and methadone); as previously discussed, access to MOUD can support sustained recovery and APNCU. While these results are positive, research has also documented psychological harms experienced during community supervision and court diversion [55, 56]. Future research should carefully examine their impacts on prenatal care utilization and pregnancy outcomes, as well as explore opportunities to translate effective ingredients into non-punitive, community-based, care-oriented alternatives to criminalization.

### Limitations

First, this study only reflects biopsychosocial correlates of prenatal care utilization for women who are connected to care (e.g., had at least 1 prenatal care visit). Second, providers at partner sites did not consistently obtain medical records for transferred patients (including those formerly incarcerated), meaning prior prenatal care may have been incompletely documented. The study team made every effort to obtain medical records; however, this study may under-detect the true prevalence of timely and adequate prenatal care for transferred patients. Third, providers incompletely documented social determinants of health. In the absence of clear guidelines on screening and documenting criminal legal status in medical records, women with CL-OUD were likely missed for inclusion. We also cannot assert a temporal relationship between social determinants and prenatal care utilization. Fourth, we were unable to cleanly isolate effects of distinct criminal legal experiences during the prenatal course due to the cross-sectional nature of the data. Fifth, our sample was drawn from a majority White region and does not represent the experiences of Black and Brown women, who nationally are disproportionately targeted by the criminal legal system. Future research should examine APNCU among racially minoritized women with CL-OUD. Finally, the Kotelchuck Index was developed based on prenatal care experiences of the general population, not women with biopsychosocial portraits and structural inequities similar to our sample. While the Kotelchuck Index is a standard surveillance measure of APNCU in the United States, its definition of adequate care may not be appropriate for women with CL-OUD. Future research should continue to characterize the pregnancy risks and care needs of women with CL-OUD and develop guidance on care plans to meet their unique needs.

## Conclusion

These results suggest that pregnant women with CL-OUD in NNE experience significant disparities in prenatal care utilization relative to the general population and that their biopsychosocial experiences are not well-explored by prenatal care providers; these are missed opportunities to improve maternal and child health. Potential facilitators of timely and adequate prenatal care for women with CL-OUD may include policy and health system efforts to expand access to MOUD treatments in rural regions, improve care engagement for patients with comorbid conditions, increase availability of rurally-based prenatal care, increase access to stable housing and social support, and develop community-based alternatives to incarceration. Additionally, health systems and practices should examine and contend with their structural biases against patients with criminal legal involvement. To this end, initiatives to advance prenatal care for women with CL-OUD include 1) improve training for prenatal care providers about substance use conditions and the health impacts of criminal legal involvement, 2) establish professional guidelines and processes for full assessment and documentation of substance use disorders and biopsychosocial contexts (e.g., American Society of Addiction Medicine criteria) for prenatal care planning, 3) develop “reach-in” strategies to the criminal legal system to improve care transitions, 4) develop guidelines and process strategies to improve care continuity and consistent medical record documentation during community prenatal care transfers; and 5) integrate social resources into prenatal and primary care to support care utilization by pregnant and parenting women (e.g., transportation, Housing First programs, medical-legal partnerships). Such initiatives should precede and prioritize the first and second trimesters, the loci of greatest prenatal care disparity in our sample.

## Data Availability

The datasets used and/or analyzed during the current study are available from Drs. Sarah Lord and Daisy Goodman on reasonable request.

## Abbreviations

ACOG: American College of Obstetrics and Gynecology
APNCU: Adequacy of prenatal care utilization
CL-OUD: Criminal legal involvement and opioid use disorder
MOUD: Medication for opioid use disorder
NNE: Northern New England
OUD: Opioid use disorder

## References

[1] Collins A, Laflamme D. Annual New Hampshire Report on Maternal Mortality. 2020. http://www.gencourt.state.nh.us/statstudcomm/committees/72/documents/2020%20Annual%20New%20Hampshire%20Report%20on%20Maternal%20Mortality%20(12%20pages).pdf. Accessed 30 Oct 2021.

[2] CDC/DHHS. Maternal, Fetal, and Infant Mortality Review Panel Annual Report. 2019. https://www.maine.gov/dhhs/sites/maine.gov.dhhs/files/documents/Maternal-Fetal-and-Infant-Mortality-Review-Panel-2019-Report-011020.pdf. Accessed 1 Nov 2022.

[3] Ko JY. Incidence of Neonatal Abstinence Syndrome — 28 States, 1999–2013. MMWR Morb Mortal Wkly Rep. 2019;65(31):799–802. 10.15585/MMWR.MM6531A2. Accessed 30 Oct 2021. 10.15585/mmwr.mm6531a2 27513154

[4] Kang-Brown J. Incarceration Trends in Maine. 2019. https://www.vera.org/publications/state-incarceration-trends/maine. Accessed 10 Sept 2022.

[5] Kang-Brown J. Incarceration Trends in New Hampshire. 2019. https://www.vera.org/publications/state-incarceration-trends/new-hampshire. Accessed 10 Sept 2022.

[6] Kang-Brown J. Incarceration Trends in Vermont. 2019. https://www.vera.org/publications/state-incarceration-trends/vermont. Accessed 10 Sept 2022.

[7] Joudrey PJ, Edelman EJ, Wang EA (2019). Drive Times to Opioid Treatment Programs in Urban and Rural Counties in 5 US States. JAMA.

[8] Villapiano NLG, Winkelman TNA, Kozhimannil KB, Davis MM, Patrick SW (2017). Rural and Urban Differences in Neonatal Abstinence Syndrome and Maternal Opioid Use, 2004 to 2013. JAMA Pediatr.

[9] Paltrow LM, Flavin J (2013). Arrests of and Forced Interventions on Pregnant Women in the United States, 1973–2005: Implications for Women’s Legal Status and Public Health. J Health Polit Policy Law.

[10] Guttmacher Institute. Substance Use During Pregnancy. 2023. https://www.guttmacher.org/state-policy/explore/substance-use-during-pregnancy. Accessed 3 Mar 2023.

[11] Miranda L, Dixon V, Reyes C. How States Handle Drug Use During Pregnancy. ProPublica. 2015. https://projects.propublica.org/graphics/maternity-drug-policies-by-state. Accessed 3 Mar 2023.

[12] Gateway CWI. Parental Substance Use as Child Abuse. 2020. https://www.childwelfare.gov/pubpdfs/parentalsubstanceuse.pdf. Accessed 3 Mar 2023.

[13] Association AB. Collateral Consequences of Criminal Convictions: Judicial Bench Book. 2018. https://www.ojp.gov/pdffiles1/nij/grants/251583.pdf. Accessed 10 Sept 2022.

[14] Faherty LJ, Stein BD, Terplan M. Consensus guidelines and state policies: the gap between principle and practice at the intersection of substance use and pregnancy. AJOG MFM. 2020. 10.1016/j.ajogmf.2020.100137 PMC7571448 33089133

[15] O’Rourke-Suchoff D, Sobel L, Holland E, Perkins R, Saia K, Bell S (2020). The labor and birth experience of women with opioid use disorder: A qualitative study. Women and Birth: Journal of the Australian College of Midwives.

[16] Kozhimannil KB, Chantarat T, Ecklund AM, Henning-Smith C, Jones C (2019). Maternal Opioid Use Disorder and Neonatal Abstinence Syndrome Among Rural US Residents, 2007–2014. J Rural Health.

[17] Sue K. Getting wrecked: Women, Incarceration, and the American Opioid Crisis (Borofskym Robert, Ed.; 1st ed.). University of California Press. 2019.

[18] Sufrin C. Jailcare: Finding the Safety Net for Women behind Bars (1st ed.). University of California Press. 2017.https://www.ucpress.edu/book/9780520288683/jailcare

[19] Mascola MA, Borders AE, Terplan M, ACOG Committee on Obstetric Practice. Opioid Use and Opioid Use Disorder in Pregnancy. ACOG. 2017;711. https://www.acog.org/clinical/clinical-guidance/committee-opinion/articles/2017/08/opioid-use-and-opioid-use-disorder-in-pregnancy. Accessed 1 Nov 2021.

[20] Sutter MB, Gopman S, Leeman L (2017). Patient-centered Care to Address Barriers for Pregnant Women with Opioid Dependence. Obstetrics and Gynecology Clinics.

[21] Dumont DM, Wildeman C, Lee H, Gjelsvik A, Valera P, Clarke JG. Incarceration, Maternal Hardship, and Perinatal Health Behaviors. Maternal Child Health J. 2014;18:9; 2179–2187. 10.1007/S10995-014-1466-3. 10.1007/s10995-014-1466-3 PMC4161663 24615355

[22] Bell J, Zimmerman F, Huebner C, Cawthon M, Ward D, Schroeder C (2004). Perinatal health service use by women released from jail. J Health Care Poor Underserved.

[23] Robbins C, Martocci S (2020). Timing of Prenatal Care Initiation in the Health Resources and Services Administration Health Center Program in 2017. Ann Intern Med.

[24] Miller M, Clarke L, Albrecht S, Farmer F (1996). The interactive effects of race and ethnicity and mother’s residence on the adequacy of prenatal care. J Rural Health.

[25] Clemans-Cope L, Lynch V, Howell E, Hill I, Holla N, Morgan J, Johnson P, Cross-Barnet C, Thompson J (2019). Pregnant women with opioid use disorder and their infants in three state Medicaid programs in 2013–2016. Drug Alcohol Depend.

[26] Harris PA, Taylor R, Thielke R, Payne J, Gonzalez N, Conde JG (2009). Research electronic data capture (REDCap)—A metadata-driven methodology and workflow process for providing translational research informatics support. J Biomed Inform.

[27] Harris PA, Taylor R, Minor BL, Elliott V, Fernandez M, O’Neal L, McLeod L, Delacqua G, Delacqua F, Kirby J, Duda SN. The REDCap consortium: Building an international community of software platform partners. J Biomedical Informatics. 2019;95. 10.1016/J.JBI.2019.103208. 10.1016/j.jbi.2019.103208 PMC7254481 31078660

[28] Kotelchuck M (1994). An evaluation of the Kessner Adequacy of Prenatal Care Index and a proposed Adequacy of Prenatal Care Utilization Index. Am J Public Health.

[29] American Academy of Pediatrics, & American College of Obstetricians and Gynecologists. Guidelines for Perinatal Care (S. J. Kilpatrick & L.A. Papile, Eds.; 8th ed.). AAP & ACOG. 2017.

[30] Yan J (2017). The Effects of Prenatal Care Utilization on Maternal Health and Health Behaviors. Health Econ.

[31] Chowdhury MZI, Turin TC. Variable selection strategies and its importance in clinical prediction modelling. Fam Med Commun Health. 2020;8(1). 10.1136/FMCH-2019-000262 10.1136/fmch-2019-000262 PMC7032893 32148735

[32] StataCorp. Stata Statistical Software: Release 16. StataCorp LLC. 2019.

[33] March of Dimes. Distribution of prenatal care timing categories: United States, 2020. Peristats Data: Prenatal Care. 2020. https://www.marchofdimes.org/peristats/data?reg=99&top=5&stop=20&lev=1&slev=4&obj=3&sreg=99. Accessed 17 Oct 2022.

[34] Osterman MJK, Martin JA. Timing and adequacy of prenatal care in the United States, 2016. National Vital Statistics Reports. 2018;67(3). https://stacks.cdc.gov/view/cdc/55174. 29874159

[35] Krans E, Kim J, Chen Q, Rothenberger S, James A, Kelley D, Jarlenski M (2021). Outcomes associated with the use of medications for opioid use disorder during pregnancy. Addiction.

[36] Schiff D, Nielsen T, Hoeppner B, Terplan M, Hadland S, Bernson D, Greenfield S, Bernstein J, Bharel M, Reddy J, Taveras E, Kelly J, Wilens T. Methadone and buprenorphine discontinuation among postpartum women with opioid use disorder. Am J Obstetr Gynecol. 2021;225(4). 10.1016/J.AJOG.2021.04.210. 10.1016/j.ajog.2021.04.210 PMC8492487 33845029

[37] Goodman DJ, Saunders EC, Wolff KB (2020). In their own words: A qualitative study of factors promoting resilience and recovery among postpartum women with opioid use disorders. BMC Pregnancy Childbirth.

[38] Amiri S, McDonell MG, Denney JT, Buchwald D, Amram O (2021). Disparities in Access to Opioid Treatment Programs and Office-Based Buprenorphine Treatment Across the Rural-Urban and Area Deprivation Continua: A US Nationwide Small Area Analysis. Value in Health.

[39] Gregory HM, Hill VM, Parker RW. Implications of Increased Access to Buprenorphine for Medical Providers in Rural Areas: A Review of the Literature and Future Directions. Cureus. 2021;13(11). 10.7759/CUREUS.19870. 10.7759/cureus.19870 PMC8712194 34976492

[40] Kumar R, Viswanath O, Saadabadi A. Buprenorphine. StatPearls. 2023. https://www.ncbi.nlm.nih.gov/books/NBK459126/. Accessed 28 Mar 2023.

[41] Lamont K, Scott NW, Gissler M, Gatt M, Bhattacharya S (2022). Risk of Recurrent Stillbirth in Subsequent Pregnancies. Obstet Gynecol.

[42] Getahun D, Fassett MJ, Jacobsen SJ (2010). Gestational diabetes: risk of recurrence in subsequent pregnancies. Am J Obstet Gynecol.

[43] Bernardes TP, Mol BW, Ravelli ACJ, van den Berg P, Marike Boezen H, Groen H. Early and late onset pre-eclampsia and small for gestational age risk in subsequent pregnancies. PLOS ONE. 2020;15(3): e0230483. 10.1371/JOURNAL.PONE.0230483 10.1371/journal.pone.0230483 PMC7100959 32218582

[44] Centers for Medicare and Medicaid Services. Improving Access to Maternal Health Care in Rural Communities. n.d. https://www.cms.gov/About-CMS/Agency-Information/OMH/equity-initiatives/rural-health/09032019-Maternal-Health-Care-in-Rural-Communities.pdf. Accessed Oct 2022.

[45] Joyce NR, Zullo AR, Ahluwalia JS, Pfeiffer MR, Curry AE (2019). Driver’s License Suspension Policies as a Barrier to Health Care. Am J Public Health.

[46] Holcomb D S, Pengetnze Y, Steele A, Karam A, Spong C, Nelson DB Geographic barriers to prenatal care access and their consequences. Am J Obstetr Gynecol MFM. 2021;3(5). 10.1016/J.AJOGMF.2021.100442 10.1016/j.ajogmf.2021.100442 34245930

[47] Maldonado LY, Fryer KE, Tucker CM, Stuebe AM (2020). The Association between Travel Time and Prenatal Care Attendance. Am J Perinatol.

[48] Gunn AJ, Sacks TK, Jemal A (2018). “That’s not me anymore”: Resistance strategies for managing intersectional stigmas for women with substance use and incarceration histories. Qual Soc Work.

[49] Gunn A, Miranda Samuels G (2020). Promoting Recovery Identities Among Mothers with Histories of Addiction: Strategies of Family Engagement. Fam Process.

[50] Scorsone KL, Haozous EA, Hayes L, Cox KJ (2021). Ending the Chase: Experiences of Rural Individuals with Opioid Use Disorder. Subst Use Misuse.

[51] WellSense Health Plan. Rides to Appointments. 2022. https://www.wellsense.org/members/nh/new-hampshire-medicaid/transportation. Accessed 21 Nov 2022.

[52] Rezansoff SN, Moniruzzaman A, Clark E, Somers JM. Beyond recidivism: changes in health and social service involvement following exposure to drug treatment court. Substance Abuse Treatment Prevention Policy. 2015;10(1). 10.1186/S13011-015-0038-X 10.1186/s13011-015-0038-x PMC4628391 26520393

[53] Kennedy-Hendricks A, Bandara S, Merritt S, Barry CL, Saloner B. Structural and organizational factors shaping access to medication treatment for opioid use disorder in community supervision. Drug Alcohol Dependence. 2021;226. 10.1016/J.DRUGALCDEP.2021.108881 10.1016/j.drugalcdep.2021.108881 34218008

[54] Chapter 2: Lawful Employment and Notification of Change in Employment (Probation and Supervised Release Conditions). 2016. https://www.uscourts.gov/services-forms/lawful-employment-notification-change-employment-probation-supervised-release-conditions. Accessed 30 Mar 2022.

[55] Ellis JD, Grekin ER, Resko SM (2019). Correlates of substance use in pregnant women under community supervision after conviction for a criminal offence: The role of psychological distress. Crim Behav Ment Health.

[56] Sung HE. Pregnancy and drinking among women offenders under community supervision in the United States: 2004-2008. J Urban Health. 2012;89(3):500–509. 10.1007/S11524-011-9658-2 10.1007/s11524-011-9658-2 PMC3368053 22311615

